# Prevalence of osteoporosis in patients with systemic lupus erythematosus: A multicenter comparative study of the World Health Organization and fracture risk assessment tool criteria

**DOI:** 10.1016/j.afos.2020.11.001

**Published:** 2020-11-27

**Authors:** Ju-Yang Jung, Sang Tae Choi, Sung-Hoon Park, Seong-Ryul Kwon, Hyoun-Ah Kim, Sung-Soo Kim, Sang Hyon Kim, Chang-Hee Suh

**Affiliations:** a Department of Rheumatology, Ajou University School of Medicine, Suwon, Republic of Korea; b Division of Rheumatology, Department of Internal Medicine, Chung-Ang University College of Medicine, Seoul, Republic of Korea; c Department of Radiology, Ajou University School of Medicine, Suwon, Republic of Korea; d Division of Rheumatology, Department of Internal Medicine, Inha University College of Medicine, Incheon, Republic of Korea; e Division of Rheumatology, Department of Internal Medicine, Ulsan University College of Medicine, Gangneung Asan Hospital, Gangneung, Republic of Korea; f Division of Rheumatology, Department of Internal Medicine, Keimyung University College of Medicine, Daegu, Republic of Korea

**Keywords:** Systemic lupus erythematosus, Osteoporosis, Fracture, Bone mineral density, Fracture risk assessment tool

## Abstract

**Objectives:**

Osteoporosis and fracture are known complications of systemic lupus erythematosus (SLE). We assessed the prevalence and risk factors for osteoporosis in patients with SLE.

**Methods:**

A total of 155 female SLE patients were recruited retrospectively in 5 university hospitals. The bone mineral density (BMD) was measured by dual-energy X-ray absorptiometry, and the fracture risk assessment tool (FRAX) for high-risk osteoporotic fractures was calculated with and without BMD.

**Results:**

The mean age was 53.7 ± 6.8 years, and osteoporotic fractures were detected in 19/127 (15.0%) patients. The proportion of patients having a high-risk for osteoporotic fractures in the FRAX with and without BMD, and osteoporosis by the World Health Organization (WHO) criteria were 25 (16.1%), 24 (15.5%), and 51 (32.9%), respectively, and 48.0–68.6% of them were receiving treatment. On multivariate logistic analysis, nephritis (odds ratio [OR] 11.35) and cumulative dose of glucocorticoid (OR 1.1) were associated with high-risk by the FRAX with BMD, and low complement levels (OR 4.38), erythrocyte sedimentation rate (ESR) (OR 1.04), and cumulative dose of glucocorticoid (OR 1.05) were associated with osteoporosis by the WHO criteria in patients with SLE.

**Conclusions:**

Among Korean female patients with SLE, the proportion of patients having a high-risk of osteoporotic fractures by the FRAX tool was 15.5%–16.1% and the proportion of patients having osteoporosis by the WHO criteria was 32.9%. In SLE, nephritis, low level of complement, ESR, and cumulative dose of glucocorticoids may contribute to fracture risk.

## 1 Introduction

Reduced bone mass or osteoporotic fracture is one of the major complications of chronic inflammatory diseases including systemic lupus erythematosus (SLE) [[1], [2], [3]]. Several population-based studies showed a higher prevalence of fracture or osteoporosis among patients with SLE [[4], [5], [6]]. The risk in patients with SLE is 1.86–1.99-fold higher for hip fracture and 2.97-fold higher for vertebral fracture compared to the normal population [7,8]. Bone loss in SLE depends not only on conventional factors such as age and lack of physical activity, but also on the systemic inflammation, musculoskeletal symptoms, and administration of glucocorticoids [[9], [10], [11], [12]]. High disease activity, frequent flare up, renal failure, and low complement levels are known contributing factors for bone loss in SLE. Therefore, fragility fracture has been regarded as one of the major disease outcomes and is included in the damage index of SLE. Although treatment strategies to reduce the cumulative dose of glucocorticoids have been implemented, the recent data showed an increased risk of osteoporotic fractures [12,13].

The occurrence of fractures not only reduces the quality of life, but also results in disability in patients with SLE. Early detection of bone loss with proper intervention is essential to prevent osteoporotic fractures. Osteoporosis is commonly defined based on bone mineral density (BMD) criteria established by the World Health Organization (WHO), and the BMD is measured by dual-energy X-ray absorptiometry (DXA). In addition, the fracture risk assessment tool (FRAX) was developed in 2008 by a WHO task force, to assess the 10-year probability for hip and major osteoporotic fractures [14,15]. The FRAX tool calculates the probability by age, sex, weight, height, smoking history, alcohol intake, history of previous fracture, family history of fracture, glucocorticoid use, and if available, the BMD results. FRAX has been recommended for the detection of high-risk of osteoporotic fractures. However, the tool has not been validated and has been rarely evaluated for assessing the risk of osteoporotic fractures in patients with SLE [13].

We aim to compare FRAX and BMD according to the WHO criteria to determine the high-risk groups who require osteoporotic treatment, and to determine if there is a gap between the high-risk groups and the patients who are on treatments. In addition, we attempt to find factors associated with high risk of osteoporotic fractures in patients with SLE.

## 2 Methods

### 2.1 Study population

In all, 155 Korean female patients with SLE, who were ≥ 40 years old were enrolled from 5 university hospitals between January 2012 and December 2016. All patients met the 1997 American College of Rheumatology (ACR) criteria for SLE [16]. The patients who checked for BMD or thoracolumbar (T-L) spine X-ray were included, however those with malignancy, or renal and hepatic failure were excluded. Retrospectively, clinical data were collected through a medical chart review, which included age, sex, body mass index (BMI), menopausal status, hormone supplement therapy in postmenopausal women, smoking status, and laboratory results. Medication history for the treatment of SLE including use of glucocorticoids and anti-osteoporotic drugs was obtained. This study was approved by the institutional review board of Ajou University Hospital, Suwon, Korea (AJIRB-MED-MDB-15-285), Chung-Ang University Hospital, Seoul, Korea (C2015163[1621]), Inha University Hospital, Incheon, Korea (2015-09-026), Gangneung Asan Hospital, Gangneung, Korea (3-32100191-AB-N-01), and Keimyung University Hospital, Daegu, Korea (DSMC2015-12-017-007).

### 2.2 Evaluation of osteoporosis by the WHO criteria and the FRAX model

The T- and Z-scores of BMD of the lumber vertebrae (L1-4) and proximal femur were measured using the DXA (GE Lunar, Madison, WI, USA). Osteoporosis was defined as a value of the T-score that was −2.5 or less for postmenopausal women or men ≥ 50 years old, and Z-scores −2 or less for premenopausal women or men <50 years old. The precision error was 0.87% for the lumbar spine BMD and 0.93% for the femoral neck BMD. The least significant changes were 0.024 and 0.026 g/cm^2^ for the BMDs at the lumbar spine and femoral neck, respectively.

We calculated FRAX values with and without BMD using the Korean model (http://www.shef.ac.uk/FRAX/tool.aspx?country=25). The FRAX with BMD was calculated including the femur neck BMD (g/cm^2^) value. Although there are no criteria defined for low bone mass or osteoporosis in the FRAX tool, a high-risk of osteoporotic fracture was defined as a 10-year probability of ≥20% for major (spine, wrist, hip, shoulder) osteoporotic fracture or ≥ 3% for hip fracture. While the data of symptomatic fractures were collected through the medical chart review and history taking, radiologic vertebral compression fracture was detected using the T-L spine X-ray (n = 127) by the radiologist (S.H.P) [17]. In addition, age and glucocorticoids-adjusted FRAX tool was applied [18,19].

### 2.3 Clinical characteristics for risk of fracture or osteoporosis

With the baseline information, the clinical characteristics of SLE were collected by chart review and interviews. These included manifestations of SLE, laboratory findings, systemic lupus erythematosus damage index (SLEDAI), medications for SLE, and osteoporosis therapy with vitamin D and calcium supplement. Data about glucocorticoid therapy were collected as the cumulative dose and current dose (prednisolone-equivalent). Osteoporotic medications included bisphosphonates and selective estrogen receptor modulators (SERM).

### 2.4 Statistical analysis

Data of the patient characteristics, laboratory tests and BMD, and medications used were analyzed as descriptive statistics. The results are presented as means ± SD, median (interquartile range, IQR), or number of patients with the percentage. Correlations between the conventional or disease related factors, and the high-risk of osteoporotic fracture according to FRAX or osteoporosis by the WHO criteria were analyzed using Spearman’s correlation. Conditional logistic regression analysis was conducted to estimate the odds ratios (ORs) and 95% confidence intervals (CI) to assess the association between the clinical characteristics and high-risk according to the FRAX or WHO osteoporosis criteria in patients with SLE. The statistical analyses were performed with SPSS for Windows (ver 23.0; IBM, Armonk, NY, USA), and P-values < 0.05 were considered statistically significant.

## 3 Results

### 3.1 Basic characteristics of the patients

The characteristics of the patients are shown in Table 1. Mean age was 53.7 ± 6.8 years, and 113 patients (72.9%) were postmenopausal. Their mean weight was 55.4 ± 9.2 kg, mean height was 156.6 ± 5.8 cm, and mean body mass index (BMI) was 22.6 ± 3.6 kg/m^2^.

**Table 1:** Characteristics of female patients with systemic lupus erythematosus.

Variable	Mean ± SD or n (%)
Number, n	155
Age, yr	53.7 ± 6.8
Postmenopausal status, n (%)	113 (72.9)
Current smoking, n (%)	2 (1.3)
Alcohol > 3 units/day, n (%)	9 (45.8)
Weight, kg	55.4 ± 9.2
Height, cm	156.6 ± 5.8
Body mass index, kg/m^2^	22.6 ± 3.6
Disease duration, months	47.2 ± 8.0
Oral ulcer, n (%)	20 (12.9)
Skin rash, n (%)	21 (13.5)
Arthritis, n (%)	91 (58.7)
Nephritis, n (%)	20 (12.9)
Serositis, n (%)	9 (5.8)
Hematologic involvement, n (%)	31 (20)
Anti-dsDNA (+), n (%)	37 (23.9)
Low complements level, n (%)	47 (30.3)
ESR, mm/hr	21.7 ± 1.5
SLEDAI	3.5 ± 0.3
Hydroxychloroquine, n (%)	141 (91)
Glucocorticoids cumulative dose, g	8.7 ± 0.8
Glucocorticoids current dose, mg/d	3.4 ± 0.5
Immunosuppressants, n (%)	66 (42.6)
Osteoporosis therapy, n (%)	48 (31)
Duration of osteoporosis therapy, years	2 (1–4)^a^
Bisphosphonate therapy, n (%)	39 (25.2)
Vitamin D supplement, n (%)	70 (45.2)
Calcium supplement, n (%)	85 (54.8)
Osteoporotic fracture, n (%)	19/127 (15)

Values are presented as mean ± SD: standard deviations (SD) or number (%), SD: standard deviations, ANA: anti-nuclear antibody, dsDNA: double-strand deoxyribonucleic acid, ESR: erythrocyte sedimentation rate, SLEDAI: systemic lupus erythematosus disease activity index.

a Median (Interquatile range).

The disease duration of the patients with SLE was 47.2 ± 8.0 months, and 91 (58.7%) patients had arthritis, 20 (12.9%) patients had nephritis, 31 (20%) patients had hematologic involvement, and the mean SLEDAI was 3.5 ± 0.3. Cumulative dose of glucocorticoid intake was 8.7 ± 0.8 g prednisolone-equivalent, and current dose of glucocorticoids was 3.4 ± 0.5 mg/day prednisolone-equivalent. Forty-eight (31%) patients were on osteoporotic therapy, of which 39 (25.2%) patients were on bisphosphonate therapy. Nineteen (15.0%) patients had confirmed osteoporotic fractures among 127 patients who underwent X-ray of the spines.

### 3.2 Patients with high-risk of osteoporotic fracture by the FRAX and osteoporosis by the WHO criteria

Among 155 patients with SLE, 3 (1.9%) and 25 (16.1%) patients had a high risk of major (osteoporotic) and hip fracture according to the FRAX with BMD, respectively (Table 2), and 4 (2.6%) and 24 (15.5%) patients had a high risk of major and hip fracture, respectively, according to the FRAX without BMD. According to the WHO criteria, 51 (32.9%) patients had osteoporosis. Among the premenopausal patients, 1 (2.4%) and 5 (11.9%) patients had a high risk of major and hip fracture, respectively, according to the FRAX with BMD, 3 (7.1%) patients had a high risk of hip fracture according to the FRAX without BMD, and 8 (19.0%) patients had osteoporosis. Among the postmenopausal patients, 2 (1.8%) and 20 (17.7%) patients had a high risk of major and hip fracture, respectively, according to the FRAX with BMD, 4 (3.5%) and 21 (18.6%) patients had a high risk of major and hip fracture, respectively, according to the FRAX without BMD, and 43 (38.1%) patients had osteoporosis.

**Table 2:** Proportions of patients having high-risk of fracture in FRAX with and without BMD and osteoporosis by the WHO criteria among patients with systemic lupus erythematosus.

Variables (patients groups), n	High-risk in FRAX with BMD	High-risk in FRAX without BMD	Osteoporosis by the WHO criteria	P-value^a^	P-value^b^	P-value^c^
Overall (n = 155)	Major fracture: 3 (1.9%) Hip fracture: 25 (16.1%)	Major fracture: 4 (2.6%) Hip fracture: 24 (15.5%)	51 (32.9%)	< 0.001	0.002	< 0.001
Premenopausal (n = 42)	Major fracture: 1 (2.4%) Hip fracture: 5 (11.9%)	Major fracture: 0 Hip fracture: 3 (7.1%)	8 (19.0%)	0.001	< 0.001	< 0.001
Postmenopausal (n = 113)	Major fracture: 2 (1.8%) Hip fracture: 20 (17.7%)	Major fracture: 4 (3.5%) Hip fracture: 21 (18.6%)	43 (38.1%)	0.001	0.075	0.002

Values are presented as number (%).

FRAX, fracture risk assessment tool; BMD, bone mineral density; WHO, World Health Organization.

∗High-risk of osteoporotic fracture: a 10-year probability of ≥ 20% for major osteoporotic fracture, or ≥3% for hip fracture.

a FRAX with BMD vs. FRAX without BMD.

b FRAX with BMD vs. WHO criteria.

c FRAX without BMD vs. WHO criteria.

When age and glucocorticoids-adjusted FRAX tool was applied, 4 (2.6%) and 19 (12.3%) patients had a high risk of major and hip fracture according to the FRAX with BMD, respectively (Supplement Table 1). Four (2.5%) and 18 (11.6%) patients had a high risk of major and hip fracture, respectively, according to the FRAX without BMD and 51 (32.9%) patients had osteoporosis. Among the premenopausal patients, 1 (2.4%) and 5 (11.9%) patients had a high risk of major and hip fracture, respectively, according to the FRAX with BMD, 3 (7.1%) patients had a high risk of hip fracture according to the FRAX without BMD, and 8 (19.0%) patients had osteoporosis, according to the WHO criteria.

Among the overall candidates for osteoporosis treatment, who were having high-risk of fracture in FRAX and defined as osteoporosis by the WHO criteria, 12 (48%), 16 (66.7%), and 35 patients (68.6%) were on osteoporosis treatment in the group with high-risk of osteoporotic fracture according to the FRAX with BMD and without BMD criteria, and WHO osteoporosis criteria, respectively (Table 3). Nine (36.0%) and 15 (62.5%) patients were taking bisphosphonates among the patients with high-risk of osteoporotic fracture according to the FRAX with BMD and without BMD criteria respectively, and 26 (51%) patients were taking bisphosphonates among the patients with osteoporosis of the WHO criteria.

**Table 3:** Proportions of patients undergoing osteoporosis treatment in high-risk group according to the high-risk of fracture in FRAX and osteoporosis by the WHO criteria.

Variable (patient groups), n	High-risk in FRAX with BMD	High-risk in FRAX without BMD	Osteoporosis by the WHO criteria
Overall	12/25 (48%)	16/24 (66.7%)	35/51 (68.6%)
Premenopausal	4/5 (80%)	3/3 (100%)	5/8 (62.5%)
Postmenopausal	8/20 (40%)	13/21 (61.9%)	30/43 (69.8%)
Bisphosphonate therapy	9/25 (36.0%)	15/24 (62.5%)	26/51 (51%)

Values are presented as number (%).

FRAX: fracture risk assessment tool, BMD: bone mineral density, WHO: World Health Organization.

### 3.3 Associations of clinical factors with a high-risk of osteoporotic fractures by the FRAX and WHO osteoporosis criteria

A high-risk osteoporotic fracture based on the FRAX with BMD criteria correlated with cumulative dose of glucocorticoids (r = 0.3, P < 0.001) (Table 4). The need for osteoporotic treatment based on the FRAX without BMD criteria correlated with cumulative dose of glucocorticoids (r = 0.21, P = 0.008). The risk of osteoporosis by the WHO criteria correlated with hematologic manifestations (r = 0.17, P = 0.04), low complement level (r = 0.26, P = 0.001), and ESR (r = 0.27, P = 0.001).

**Table 4:** Correlation of clinical factors for high-risk of fracture in FRAX with and without BMD and osteoporosis by the WHO criteria among patients with systemic lupus erythematosus.

Variable	High-risk in FRAX with BMD (r, P-value)	High-risk in FRAX without BMD (r, P-value)	Osteoporosis by the WHO criteria (r, P-value)
Oral ulcer	−0.01, 0.88	−0.01, 0.87	−0.07, 0.42
Arthritis	0.12, 0.14	0.14, 0.08	0.01, 0.98
Hematologic manifestation	−0.09, 0.28	0.05, 0.51	0.17, 0.04∗
Serositis	−0.01, 0.88	0.05, 0.57	−0.06, 0.58
Nephritis	−0.12, 0.15	−0.06, 0.47	−0.07, 0.42
SLEDAI	−0.04, 0.6	0.01, 0.91	−0.06, 0.44
Low complement level	−0.06, 0.5	−0.12, 0.13	0.26, 0.001∗
ESR	0.13, 0.12	0.08, 0.35	0.27, 0.001∗
Glucocorticoid cumulative dose	0.3, < 0.001∗	0.21, 0.008∗	0.16, 0.05

FRAX, fracture risk assessment tool; BMD, bone mineral density; WHO, World Health Organization; SLEDAI, systemic lupus erythematosus disease activity index; ESR: erythrocyte sedimentation rate.

On multivariate logistic analysis, only cumulative dose of glucocorticoids (OR 1.1, 95% CI 1.05–1.15, P < 0.001) was associated with a high-risk of osteoporotic fractures based on the FRAX with BMD criteria (Table 5). Low complement levels (OR 4.38, 95% CI 1.5–12.81, P = 0.007), ESR (OR 1.04, 95% CI 1.02–1.07, P = 0.002), and cumulative dose of glucocorticoids (OR 1.05, 95% CI 1.01–1.09, P = 0.03) were associated with osteoporosis based on the WHO criteria.

**Table 5:** Multivariable logistic regression analysis for high-risk of fracture in FRAX with and without BMD and osteoporosis by the WHO criteria among patients with systemic lupus erythematosus

Variables	High-risk in FRAX with BMD		High-risk in FRAX without BMD		Osteoporosis by the WHO criteria	
	OR (95% CI)	P-value	OR (95% CI)	P-value	OR (95% CI)	P*-*value
Oral ulcer	0.88 (0.19, 4.13)	0.87	0.83 (0.2, 3.6)	0.85	0.91 (0.35, 3.93)	0.88
Arthritis	0.91 (0.26, 3.15)	0.91	0.4 (0.13, 1.26)	0.12	0.97 (0.41, 2.1)	0.85
Hematologic manifestation	5.72 (0.95, 34.49)	0.06	0.62 (0.18, 2.13)	0.45	0.41 (0.24, 1.58)	0.09
Serositis	NA	NA	3.0 (0.43, 20.96)	0.27	0.56 (0.2, 9.63)	0.73
Nephritis	11.35 (1.09, 118.57)	0.04∗	1.09 (0.16, 7.43)	0.82	1.59 (0.25, 4.11)	0.55
SLEDAI	0.99 (0.85, 1.15)	0.87	0.86 (0.69, 1.07)	0.19	0.88 (0.79, 1.06)	0.08
Low complement level	1.06 (0.31, 3.69)	0.93	1.86 (0.54, 6.39)	0.12	4.38 (1.5, 12.81)	0.007∗
ESR	1.01 (0.99, 1.04)	0.09	1.0 (0.98,1.04)	0.48	1.04 (1.02, 1.07)	0.002∗
Glucocorticoid cumulative dose, g	1.1 (1.05, 1.15)	<0.001∗	1.04 (1.00, 1.08)	0.05	1.05 (1.01, 1.09)	0.03∗

FRAX, fracture risk assessment tool; BMD, bone mineral density; WHO, World Health Organization; SLEDAI, systemic lupus erythematosus disease activity index; ESR: erythrocyte sedimentation rate; NA, not applicable.

### 3.4 Comparison of the clinical factors and proportions in high-risk of osteoporotic fracture by the FRAX and osteoporosis between SLE patients with and without fracture

The clinical factors were compared between SLE patients with and without fracture and there was no significant difference (Table 6). Between the groups with fracture and without fracture, 5 patients (27.8%) and 8 patients (9.2%) had a high-risk of osteoporotic fracture by the FRAX with BMD (P = 0.03), 7 patients (38.9%) and 8 patients (9.2%) had a high-risk of osteoporotic fracture by the FRAX without BMD (P = 0.001), and 9 patients (50.0%) and 24 patients (27.6%) had osteoporosis by WHO criteria (P = 0.06).

**Table 6:** Comparison of clinical factors between SLE patients with fracture and those not.

Variable	Fracture (n = 18)	No fracture (n = 87)	P-value
Age, yr	53.8 ± 8.6	49.1 ± 10.7	0.22
Postmenopause, n (%)	14 (77.8)	62 (71.3)	0.58
Weight, kg	56.6 ± 10.6	55.8 ± 8.1	0.52
Height, cm	155.3 ± 6.3	157.6 ± 5.7	0.21
BMI, kg/m^2^	23.4 ± 3.7	22.5 ± 3.3	0.18
Arthritis, n (%)	10 (55.6)	53 (60.9)	0.67
Nephritis, n (%)	2 (11.1)	8 (9.2)	0.8
Low complement, n (%)	3 (17.6)	25 (28.7)	0.35
ESR, mm/h	16.2 ± 10	18.2 ± 15.2	0.97
Glucocorticoid current dose, g	1.5 ± 2	3.8 ± 7.3	0.06
Glucocorticoid cumulative dose, g	7.9 ± 8.4	8.1 ± 11	0.71
High-risk in the FRAX with BMD, n (%)	5 (27.8)	8 (9.2)	0.03
High-risk in the FRAX without BMD, n (%)	7 (38.9)	8 (9.2)	0.001
Osteoporosis by the WHO criteria, n (%)	9 (50)	24 (27.6)	0.06

Values are presented as mean ± standard deviations (SD) or number (%).

SLE, systemic lupus erythematosus; BMI, body mass index; ESR, erythrocyte sedimentation rate; FRAX, fracture risk assessment tool; BMD, bone mineral density; WHO, World Health Organization.

∗Data was collected from only 105 patients who had a history of fracture or X-ray results of thoracolumbar spine.

## 4 Discussion

This study evaluated the high risk of osteoprotic fracture as defined by the FRAX model and the risk of osteoporosis based on the WHO criteria in patients with SLE. The proportion of patients having high-risk for fractures in the FRAX with BMD and without BMD and WHO criteria were 16.1, 15.5% and 32.9%, respectively. Among the patients having high-risk for fractures in the FRAX and the WHO criteria, 48–68.6% were taking preventive therapy including bisphosphonates. On multivariate logistic analysis, low complement levels, ESR, and cumulative dose of glucocorticoids were associated with osteoporosis in the WHO criteria.

The proportion of patients with high-risk of fracture based on the FRAX tool was about 2-fold smaller than that of those having osteoporosis in the WHO criteria in patients with SLE. The parameters used in calculation of the FRAX model were age, weight, history of previous fracture, family history, current smoking history, use of steroid, and history of alcohol comsumption, while osteoporosis was determined by T or Z-score from the DXA. The FRAX results, calculated from the clinical features of patients with SLE and BMD results from the DXA were different, and more patients with SLE were classified as candidates for osteoporotic treatment based on BMD according to the WHO criteria. The tool using typical risk factors may be limited in predicting a fracture or in assessing bone health in patients with SLE. This suggests that the status of bone mass represented by BMD may be poorer than the clinical status of patients with SLE.

There were some data from investigations regarding the risk of fracture using FRAX tool in patients with SLE. Among 271 Canadian women with SLE, the proportion of patients with high-risk of FRAX tool was 5.3% (major) and 9.4% (hip), while osteoporosis of the WHO criteria was defined in 14.6% [20]. As similar as our data, it also showed that the proportion of high-risk of fracture risk using FRAX tool was lower than those with osteoporosis of the WHO criteria. A study with 45 Asian patients with SLE showed that 16% was described as having high-risk of FRAX tool, and the associated factors to high-risk of FRAX tool were age and cumulative dose of glucocorticoids (major), and age and serum anti-dsDNA (hip) [21].

About 50% of the patients with a high-risk of fracture using the FRAX tool and the WHO criteria were taking medication for osteoporosis. That may be due to poor adherence to osteoporosis medication including bisphosphonates. A previous report showed that compliance with the osteoporosis medication is low, especially in the first year, and the compliance of bisphosphonates ranged from 17.7% to 74.8% [22,23]. Loss of bone mass develops without any symptoms, and patients do not think that it is necessary to take preventive medication to prevent fractures in the future [24,25]. A number of patients with SLE may ignore the risk despite the poor results of BMD, or develop adverse effects from the osteoporotic drugs, such as gastritis due to bisphosphonates or edema due to SERMs. Most patients with SLE were already taking several drugs for SLE, and reluctant to take more medications.

The causes of bone loss in SLE have not been well understood, and are considered to be multifactorial. Chronic inflammation, hormonal factors, and glucocorticoids have been found to be associated with bone loss or fracture [[26], [27], [28], [29], [30]]. Low complement levels and ESR were associated with osteoporosis based on the WHO criteria, but this was not seen in the FRAX criteria. Low levels of complements suggest a higher disease activity of SLE; thus, an active disease may contribute to low bone mass in SLE. In the previous data, anti-dsDNA antibody was associated with a high-risk of fractures by the FRAX model in patients with SLE [21]. Interestingly, lupus nephritis was associated with high-risk of fractures in the FRAX with BMD, but not with osteoporosis of the WHO criteria. Impaired kidney function represented as increased serum creatinine levels was associated with the BMD result in women with SLE, and secondary hyperparathyroidism, activated osteoclastic bone resorption, and vitamin D deficiency may contribute to bone loss [11]. Nephritis is one of the severe manifestations in SLE, and suggests the active status. Patients with lupus nephritis may have higher risk of low bone mass or fractures than those who do not.

The use of glucocorticoids is a well-known risk factor for fractures, and long-term use of glucocorticoids is the main contributor of low bone mass in SLE patients [[31], [32], [33]]. In this study, the cumulative dose of glucocorticoids was associated with a high-risk of osteoporotic fracture based on the FRAX with/without BMD criteria as well as the osteoporosis by the WHO criteria. These results confirm that low bone mass or risk of osteoporotic fractures can develop despite a low dose of glucocorticoids being taken by the patients in this study (current dose of glucocorticoids- 3.4 ± 0.5 mg/day, prednisolone-equivalent) [33].

This study has some limitations. There could be a selection bias because it is a retrospective study and the data were collected from the patients who had undergone BMD. The BMD test for premenopausal women may have been conducted because they were on glucocorticoids or had low body weight. The presence of osteoporotic fractures of 57 patients was unknown, because the timing of the T-L spine X-ray exam differed from that of the BMD. Also, the duration or type of osteoporosis therapy may affect BMD, but there was no significant finding due to the small number of the patients with osteoporosis therapy and their various durations.

The prevalences of high-risk of osteoporotic fractures based on the FRAX or osteoporosis by the WHO criteria was not compared with healthy controls, although such epidemiologic information has been reported in a previous study comprising a large cohort or using data from the national health database [34]. A study using the Korean National Health Insurance Service database showed a 2.96-fold higher risk of osteoporotic fractures in patients with SLE compared to controls [12].

This multicenter study evaluated the prevalence of high-risk of fractures based on the FRAX criteria and osteoporosis based on the WHO criteria in patients with SLE, and determined clinical features related to fracture risk. Significantly more number of patients had ostoeoporosis according to the WHO criteria compared to those with a high-risk of fracture according to the FRAX criteria. Among them, 48–68.6% of the patients were on osteoporotic treatment. Among some clinical factors, nephritis, low complement levels, ESR, and cumulatedglucocorticoid dose correlated with osteoporosis based on the WHO criteria in patients with SLE, and the clinical features related with SLE were not associated with radiologic vertebral compression fracture.

## 5 Conclusions

Among 155 female patients with SLE, 25 (16.1%) and 24 (15.5%) patients were at a high-risk of osteoporotic fractures by the FRAX with and without BMD criteria, respectively, while 51 (32.9%) patients were diagnosed with osteoporosis by the WHO criteria. Osteoporotic fractures were detected in 19/127 (15.0%) patients. Nephritis and cumulative dose of glucocorticoid were associated with high-risk by the FRAX with BMD, and low complement levels, ESR, and cumulative dose of glucocorticoid were associated with osteoporosis by the WHO criteria in patients with SLE.

## CRediT author statement

**Ju-Yang Jung:** Conceptualization, formal analysis, investigation, writing-original draft.

**Sang Tae Choi:** Conceptualization, investigation, resources.

**Sung-Hoon Park:** Methodology, visualization.

**Seong-Ryul Kwon:** Conceptualization, investigation, resources.

**Hyoun-Ah Kim:** Conceptualization, investigation, resources**.**

**Sung-Soo Kim:** Conceptualization, investigation, resources.

**Sang Hyon Kim:** Conceptualization, investigation, resources.

**Chang-Hee Suh:** Conceptualization, formal analysis, investigation, writing-review & editing, project administration, funding acquisition.

## Conflicts of interest

The authors declare no competing interests.
